# The Effect of Reminiscence Therapy Using Virtual Reality on Apathy in Residential Aged Care: Multisite Nonrandomized Controlled Trial

**DOI:** 10.2196/29210

**Published:** 2021-09-20

**Authors:** Dimitrios Saredakis, Hannah AD Keage, Megan Corlis, Erica S Ghezzi, Helen Loffler, Tobias Loetscher

**Affiliations:** 1 UniSA Justice & Society University of South Australia Adelaide, South Australia Australia; 2 UniSA Clinical & Health Sciences University of South Australia Adelaide, South Australia Australia; 3 Helping Hand Aged Care Adelaide, South Australia Australia

**Keywords:** reminiscence, head-mounted display, apathy, cognitive aging, dementia, residential facilities, virtual reality

## Abstract

**Background:**

Apathy is a frequent and underrecognized neurological disorder symptom. Reduced goal-directed behavior caused by apathy is associated with poor outcomes for older adults in residential aged care. Recommended nonpharmacological treatments include person-centered therapy using information and communication technology. Virtual reality (VR) in the form of head-mounted displays (HMDs) is a fully immersive technology that provides access to a wide range of freely available content. The use of VR as a therapy tool has demonstrated promise in the treatment of posttraumatic stress disorder and anxiety. In addition, VR has been used to improve conditions including depression, anxiety, cognitive function, and balance in older adults with memory deficits, Alzheimer disease, and Parkinson disease. Research using VR for the symptoms of apathy in older adults living in residential aged care facilities is limited.

**Objective:**

This study aims to examine whether using HMDs as a tool for reminiscence therapy improves the symptoms of apathy compared with using a laptop computer and physical items with older adults living in residential aged care.

**Methods:**

In this multisite trial, 43 participants were allocated to one of three groups: reminiscence therapy intervention using VR in the form of HMDs, reminiscence therapy using a laptop computer supplemented by physical items if required (active control), and a usual care (passive control) group. The primary outcome was apathy, and the secondary outcomes included cognition and depression. The side effects of using HMDs were also measured in the VR group.

**Results:**

Mixed model analyses revealed no significant group interaction over time in outcomes between the VR and laptop groups (estimate=−2.24, SE 1.89; *t*_40_=−1.18; *P=*.24). Pooled apathy scores in the two intervention groups compared with the passive control group also revealed no significant group interaction over time (estimate=−0.26, SE 1.66; *t*_40_=−0.16; *P=*.88). There were no significant secondary outcomes. Most participants in the VR group stated that they would prefer to watch content in VR than on a flat screen (*Χ*^2^_2_=11.2; *P*=.004), side effects from HMD use were negligible to minimal according to the Simulator Sickness Questionnaire cutoff scores.

**Conclusions:**

Although there were no significant results in outcome measures, this study found that participants engaged in the research and enjoyed the process of reminiscing using both forms of technology. It was found that VR can be implemented in an aged care setting with correct protocols in place. Providing residents in aged care with a choice of technology may assist in increasing participation in activities. We cannot dismiss the importance of immediate effects while the therapy was in progress, and this is an avenue for future research.

**Trial Registration:**

Australian New Zealand Clinical Trials Registry ACTRN12619001510134; https://www.anzctr.org.au/Trial/Registration/TrialReview.aspx?id=378564.

**International Registered Report Identifier (IRRID):**

RR2-DOI: 10.1136/bmjopen-2020-046030

## Introduction

### Background

#### Apathy

Apathy is a neurological disorder with a high prevalence found in older adults living in residential aged care facilities [1,2]. Characterized as a lack of interest and diminished motivation [3], the presence of apathy results in reduced goal-directed behavior [4,5], which can result in withdrawal from activities. In long-term residential aged care facilities, lower engagement in activities and social interaction is associated with poorer quality of life [6].

Prognostic features of apathy include accelerated cognitive decline [7], and in aged care, apathy has been associated with an increased risk of mortality [8]. Although apathy is not as disruptive as other neuropsychiatric symptoms, including aggression and agitation, it can be a predictor of caregiver burden [9]. There is a lack of convincing evidence for pharmacological interventions for apathy [10-12]. Therefore, nonpharmacological individualized interventions are recommended [13,14].

#### Reminiscence Therapy

One such approach that can be individualized and tailored to a person is reminiscence therapy [15]. Commonly used in residential aged care facilities, reminiscence is a strength-based, person-centered approach that is recommended for consideration in clinical practice when caring for people with dementia [16]. The process of reminiscence involves recalling memories through the experience and discussion of past events and is commonly done with the assistance of familiar items [17]. The focus is on early memories. Reminiscence approaches can be classified into three types [18]. Simple reminiscence involves recalling positive memories to increase positive feelings [19]. Life review aims to consolidate both positive and negative previous life events into a meaningful life story, and finally, life review therapy involves redefining the negative interpretations of a person’s past [19]. It is possible that the recall of both positive and negative memories may cause distress [20,21]; however, no reported harm has been found in a review examining reminiscence therapy [17]. In this study, a semistructured simple reminiscence approach was used, focusing on positive memories.

Positive outcomes of reminiscence found in people with Alzheimer disease include improvements in cognition, depression, and quality of life [22]. Results from a meta-analysis have reported medium effects for depression (*g*=0.57, 95% CI 0.44 to 0.70) and small effects for overall mental health symptoms (*g*=0.33, 95% CI 0.16 to 0.51) [18]. The retrieval of autobiographical memories through reminiscence also provides the social function of sharing memories with other people [23].

Autobiographical memories are an important aspect of the sense of self [24], which can be impaired in those with apathy [25,26]. In aged care, the sharing of memories or life stories can positively influence the sense of self for people with dementia [27]. Therefore, reliving autobiographical memories may translate into improvements in apathy. Although reminiscence can be performed in a group or individually, it has been recommended that individual approaches may provide increased benefits [28]. An individual approach allows sessions to be tailored specifically to a person by focusing on appropriate and relevant memories depending on the person’s background. Apathy can cause a lack of interest in participation in traditional forms of activities or therapy and places the person at risk of loneliness, a prevalent problem in aged care [29]. Therefore, alternative solutions for treating apathy are required. Technology provides easy access to content and assists in maintaining engagement in therapy [15,30]. The use of immersive technology may increase therapeutic outcomes.

#### Virtual Reality

Technology can provide different levels of immersion, for example, computers or tablets are described as nonimmersive, and large screen televisions or projectors are semi-immersive [31]. An example of highly immersive virtual reality (VR) technology is the cave automatic virtual environment, where the image is displayed on the walls, and in some cases, on the floor and ceiling; however, this system is expensive, takes time to set up, and requires a dedicated room [32]. Another example of highly immersive VR technology is the head-mounted display (HMD); modern HMDs consist of a monitor for each eye to provide a stereoscopic image via a device worn on the head [33]. Available applications for HMDs can be passive, for example, providing a 360° view of a scenic virtual environment that changes as the viewer turns their head [34]. Applications can also allow users to pick up objects or physically move in a virtual environment, providing an interactive experience [34].

The successful use of HMDs as a tool for interventions includes exposure and distraction-based therapies [35,36]. Being a fully immersive technology, VR using HMDs eliminates any distractions and can increase the sense of presence, the psychological feeling of being in the virtual environment [37]. A high sense of presence may increase the response to the virtual environment [38]. For example, a study consisting of a sample of young adults found that autobiographical memory improved when content was viewed in VR using HMDs compared with a flat screen monitor [39]. Similar results of improved memory performance were found in participants who used HMDs compared with a desktop display in a sample of participants from a university campus and community [40]. Using HMDs may provide increased stimulation in the process of reliving autobiographical memories in older clinical populations because of the use of a fully immersive and realistic environment. Whether this translates to improved outcomes for older adults living in residential aged care is uncertain.

Two recent reviews have reported that VR HMD applications demonstrated potential for rehabilitation in neurological diseases, including dementia, Parkinson disease, multiple sclerosis, and stroke, or of those with pain and memory deficits [41,42]. Positive outcomes, ranging from small to large effects, found in the review by Dermody et al [42], included improved pain scores in community-dwelling older adults using VR games (*d*=−1.54) [43], and improvements in long-term recall (*d*=0.70) using VR memory training in aged care residents [44]. However, no significant outcomes were reported in psychological health, activities of daily living, depression, and social life in the studies included in this review [43,45]. A recent study of 236 older adults from community centers used familiar VR content to stimulate memory. This study found significant increases in positive affect scores (mean difference 2.09) and decreases in negative affect scores (mean difference −1.99) [46]. There is evidence that using VR with HMDs with older adults can be successful [42]; however, there is a lack of comparison between VR and flat screen technology to establish the differences and advantages of using VR.

There is a paucity of evidence comparing nonimmersive or flat screen technology with fully immersive technology (HMDs) in a therapeutic context in clinical older populations. A single-session study in aged care residents compared viewing Google Maps Street View using a tablet and HMD [47]. This study found that both forms of technology provided emotional experiences for older adults. In addition, none of the 7 participants preferred using HMD over the tablet. However, an exercise study in aged care residents reported an increase in interest and enjoyment when using HMDs compared with when watching a flat screen [48]. In another study, greater improvements in physical well-being were seen in older adults in an assisting living community when viewing content related to their past, travel, and relaxation using VR as compared with television [49].

Exposure to virtual environments can cause side effects or after effects, including eyestrain, nausea, and headaches [50,51]. This can have health and safety implications when using HMDs. Therefore, there is a need to increase our understanding of the side effects of HMD use in older adults and clinical populations who are taking medication and have symptoms related to their condition that may exacerbate any VR side effects.

### Objectives

The primary aim of this study is to examine whether using HMDs as a tool for reminiscence therapy improved outcomes compared with using a laptop computer supplemented by physical items if required (active control group). The primary outcome was apathy. Secondary outcomes included cognition and depression. Exploratory outcomes included loneliness and quality of life. The side effects of using HMDs were also examined in the VR group. We hypothesize the following: (1) the VR group would have lower apathy scores than the active control group using a laptop computer and physical items after the intervention and (2) both the VR group and active control combined would have lower apathy scores than the passive control (usual care) group after the intervention.

## Methods

### Study Design

This was a multisite nonrandomized controlled trial. Data were collected across 3 residential aged care facilities in Adelaide, South Australia. Each site was allocated a group determined by the aged care provider. Assessors conducting baseline and follow-up measures were blinded to group allocation. Participants in the intervention group were blinded to the presence of other conditions. The study was performed between December 2019 and February 2021. The CONSORT (Consolidated Standards of Reporting Trials) 2010 statement guidelines were followed [52]. A summary of the study methods is provided below, and further details of the study methods are available in the published study protocol [53].

### Participants

Eligible participants were aged 65 years or older and included only those with up to moderate impairment according to the Psychogeriatric Assessment Scale [54] as assessed by the aged care facility. Participants had to be English speaking and willing to undertake follow-up assessments. Participants in the VR group had to be able to tolerate wearing an HMD and have vision that can be corrected using their eyeglasses. The eyeglass frame also needed to fit the HMD. Participants were excluded if their score on the Psychogeriatric Assessment Scale was higher than 15 or if they had significant disorders, conditions, or behaviors that would make assessment difficult. Those with confusion or disorientation issues and who might become distressed because of confusion regarding time and place were also excluded. Participants who were interested and met the eligibility criteria were provided written and verbal information about the study. A dedicated research nurse employed by the residential aged care facility or primary researcher obtained informed consent. All participants were given the opportunity to discuss their participation with family members or other responsible persons close to the participant. Consent was continually monitored during the research by asking participants at the start and end of each session if they wanted to continue. Ethics approval was obtained from the University of South Australia Human Research Ethics Committee. We refer to the published protocol for sample size calculation [53].

### Materials

The terminology VR is an umbrella term that can refer to both nonimmersive and immersive technologies [55]; for the purposes of this study, VR will refer to immersive HMDs.

#### VR Software

YouTube VR (developed by Google LLC), a VR version of YouTube, was used to view personalized videos in VR. Wander (developed by Parkline Interactive), which uses data from Google Street View, was used to view personalized places of interest. The active control group used a laptop for viewing content; therefore, Google Street View, YouTube, and the internet in general were used to view content on the laptop.

#### VR Hardware

The Oculus Quest [56] HMD, a commercially available stand-alone headset, was used for the VR group. This HMD provides stereoscopic vision at a resolution of 1440 × 1600 per eye with a 72 Hz refresh rate and provides access to the two VR software applications used.

### Primary Outcome: Apathy Evaluation Scale Clinician Version

The Apathy Evaluation Scale (AES) clinician version, an 18-item scale, was used to measure apathy [3]. This scale also consists of an interview with the participant before completion to build rapport and gain an understanding of the participant. Each item is recorded with responses ranging from *not at all characteristic*, *slightly characteristic*, *somewhat characteristic*, and *a lot.* Four items were self-evaluated, and the remaining 14 items were assessed by the researcher. Five items assessed by the researcher need to be quantified, for example, a participant is required to give three or more examples to meet the criteria for *a lot.* Scores range from 18 to 72, with higher scores indicating increased apathy. The clinician version of the AES has a test-retest reliability of .88 and good internal consistency (α=.90) [3].

### Secondary Outcomes

#### Addenbrooke Cognitive Examination III

Addenbrooke Cognitive Examination III (ACE-III) was used to assess cognitive ability [57]. ACE-III comprises subtests of attention, memory, fluency, language, and visuospatial functioning. Its maximum score is 100, with higher scores indicating higher cognitive functioning. ACE-III reported good internal consistency (α=.88) [58].

#### Geriatric Depression Scale Short Form

The Geriatric Depression Scale (GDS) was used to measure depression [59]. This scale was developed for older populations and is suitable for those with cognitive impairment and living in long-term institutional environments [60]. The GDS has 15 items that a participant needs to respond to with a *yes* or *no* answer. Scores range from 0 to 15. A score of >5 suggests depression, whereas a score of ≥ 10 indicates depression. The GDS has 92% sensitivity and 89% specificity when compared with diagnostic criteria [60], and good internal consistency has been demonstrated (α=.80) [61].

### Exploratory Outcomes

#### The Quality of Life in Alzheimer Disease

Quality of life was measured using the Quality of Life in Alzheimer Disease (QOL-AD) 13-item scale [62]. Responses to items range from *poor, fair,* and *good* to *excellent*, with a score ranging from 13 to 52. Higher scores indicate a higher quality of life. Good internal consistency for the QOL-AD scale has been reported (α=.82) [63].

#### Three-Item Loneliness Scale

The Three-Item Loneliness Scale, a shortened version of the Revised University of California, Los Angeles Loneliness Scale [64], was used to assess the participants’ level of loneliness. Three questions were rated on a three-point scale, with scores ranging from 3 to 9. Responses range from *hardly ever* to *some of the time* to *often*. Higher scores indicated higher levels of loneliness. Acceptable internal consistency has been reported for the Three-Item Loneliness Scale (α=.72) [64].

#### Simulator Sickness Questionnaire

The Simulator Sickness Questionnaire (SSQ) was used to measure the side effects of HMD for the VR group [50]. The SSQ comprises 16 items on a four-point scale, ranging from *none, slight,* and *moderate,* to *severe.* The three subscales measure symptoms related to nausea, oculomotor, and disorientation. Higher scores indicate higher symptoms of sickness. The SSQ is the most commonly used questionnaire in VR research [65].

#### Staff Questionnaire

The Staff Questionnaire was developed by the primary researcher to measure improvement or deterioration in participants from the staff members’ point of view. Responses were reported on a five-point scale ranging from *not at all* to *very much so.* Domains measured included social involvement, cognitive awareness, pain, activities of daily living, behavior, and communication. The same domains were assessed for improvement and deterioration. Higher scores on the improvement questions meant more improvement, whereas higher scores on the deterioration questions meant more deterioration.

#### Session Record

To measure participant attendance and responses to the reminiscence sessions, a Bender session record [66] was completed for both the VR and active control groups after each reminiscence session by the researchers delivering the reminiscence content. This measure examined five subscales including *Attendance of session*, *Memory*, *Interaction*, *Responsiveness*, and *Enjoyment*. Each subscale was measured on a four-point scale ranging from 0 to 3. In addition, for both groups, participants were asked, “Would you like to do it again?” In the VR group, participants were additionally asked, “If given a choice, would you prefer to view content in VR or on a flat screen?” This was compared with the participants’ previous experience of viewing television or large screen displays.

### Procedure

#### Recruitment

**Figure 1:**
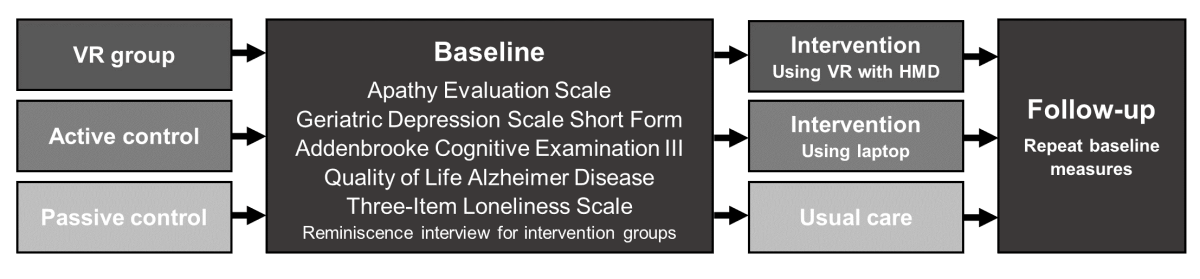
Study flow chart of procedure. HMD: head-mounted display; VR: virtual reality.

Participants were selected for recruitment by senior staff at the residential aged care facility according to the inclusion and exclusion criteria. Recruitment was undertaken by a dedicated research nurse employed by the aged care facility or the lead researcher. Participants who were interested in participating in the VR group were given a demonstration of the HMD to ensure that they could tolerate wearing it and could view images to a satisfactory standard before consent. The study flowchart is shown in Figure 1.

#### Baseline

During the baseline session, the AES, ACE-III, GDS, QOL-AD scale, and Three-Item Loneliness Scale were completed for all three groups by trained research assistants (blinded to group allocation). This process took approximately 60 minutes. For both intervention groups, a qualitative interview was conducted by a different trained researcher (unblinded to group allocation) to establish topics for the reminiscence sessions after the baseline measures were completed. This process took approximately 60 minutes. Therefore, the baseline sessions for the two intervention groups took approximately two hours. The qualitative interview was semistructured and covered themes throughout the lifespan of each participant. This included early childhood memories, adolescence, young adulthood, older adulthood, most recent memories, and musical memories. The focus was on positive experiences using reminiscence therapy guidelines [15,66].

#### Intervention

The VR and active control groups undertook three individual reminiscence sessions that were completed within a period of approximately 2 weeks at least 1 day apart. The reminiscence component was timed for 20 minutes. Additional time was taken to introduce and summarize the sessions. SSQ was completed before and after reminiscence sessions 1 and 3 for the VR group, adding approximately 10 minutes. Sessions were run by the lead researcher or a trained research assistant who was not involved in taking outcome measures. During the reminiscence sessions, there was continual conversation between the researcher and the participant regarding the content being viewed. Participants in the active control group viewed the reminiscence content on a laptop computer. The Oculus Quest HMD was used in the VR group. During the VR reminiscence experience, participants were seated in a swivel chair with arm rests when possible. If they were unable to sit in a chair, they remained in their bed; therefore, this was a passive VR experience. Content relating to the participant’s background gained from the reminiscence interview was viewed using 360° videos from YouTube VR and street view content from the Wander app. All interactions were performed by the researcher, and sound was provided by the integrated speakers of the Oculus Quest HMD. Participants were closely monitored and asked about any symptoms or side effects of viewing content in the HMD during the sessions. Content from the HMD was mirrored onto a laptop computer to enable the researcher to select content for the participant and view the content simultaneously. The passive control group did not receive the intervention and continued with their usual care during the 2 weeks.

#### Objective Measures

This study collected data on objective measures that will not be reported here. Heart rate variability, galvanic skin response, and speech measures were collected for both intervention groups during the intervention sessions, and approximately 30 minutes were added to the sessions. Activity was measured 48 hours before baseline and 48 hours after follow-up for all three groups [53].

#### Follow-up

For both intervention groups, follow-up measures were completed the day after the final reminiscence session. The average time between baseline and follow-up for participants in all three groups was approximately 2 weeks plus 4 days for the measuring of activity, as described above. For 2 participants in the passive control group, restricted site access because of COVID-19 increased the time between baseline and follow-up sessions to almost 4 weeks. None of the participants were restricted from participating in lifestyle activities offered by the aged care facility during the research period. Lifestyle activities were reduced because of COVID-19 restrictions.

### Statistical Methods

Data were entered using Research Electronic Data Capture [67] hosted by the University of South Australia. The analysis was performed using Jamovi (version 1.6.15) [68]. The primary outcome figure was created in R Studio (version 3.6.3) [69] using open visualizations [70]. The primary outcomes at baseline and follow-up were analyzed using linear mixed modeling with Helmert contrasts. This consisted of fixed factors of group (VR, active control, and passive control) and time (baseline and follow-up), with the intercept of the participant as a random factor. For comparisons of the two intervention groups with the usual care group, Helmert contrasts were used where the contrasts compared (1) combined intervention groups with the usual care group and (2) the two interventions. Additional analyses were performed by including only participants who met the criteria for a diagnosis of apathy at baseline using a cutoff score of 37.5 [3,71]. The influence of potential covariates, including depression and cognition at baseline, was examined. ACE-III, GDS, QOL-AD scale, and the Three-Item Loneliness Scale were analyzed as per the primary outcome. Assumptions of normality using the Shapiro-Wilk test found SSQ total and subscale scores to significantly deviate from a normal distribution; therefore, Wilcoxon signed-rank tests were performed for all SSQ comparisons. Assumptions of normality using the Shapiro-Wilk test found session record total and subscale scores, except for memory, to significantly deviate from a normal distribution; therefore, Mann-Whitney U tests were performed for all session record comparisons. No adjustments for multiple comparisons were made for all planned analyses; therefore, significance was set at <.05. Effect sizes for linear mixed modeling were calculated using partial η^2^ calculated from the *t* statistic and sample size with the following cutoffs: small=0.01, medium=0.06, and large=0.14.

## Results

### Demographic and Baseline Scores

**Figure 2:**
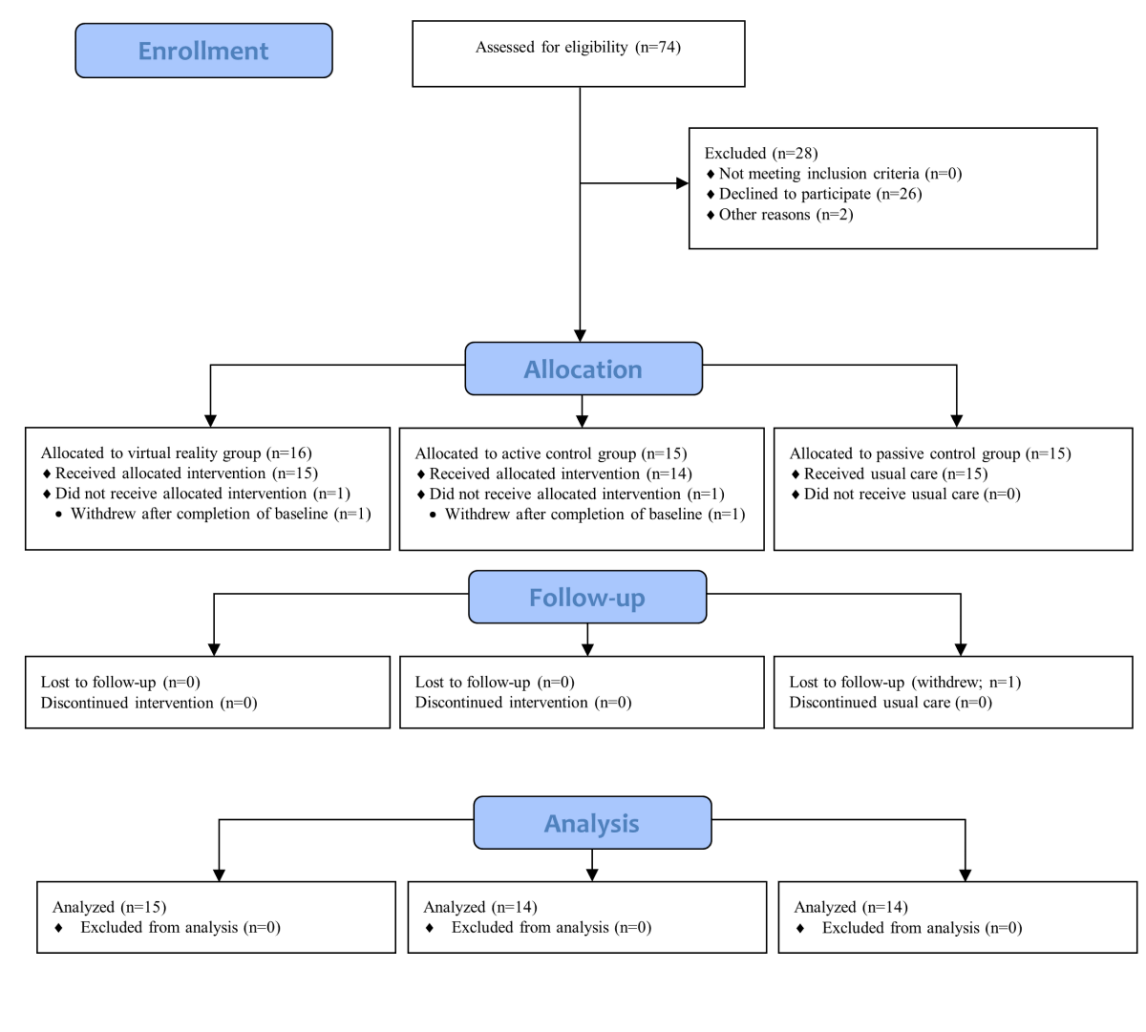
CONSORT (Consolidated Standards of Reporting Trials) flow diagram of study enrollment and analyses (modified for nonrandomized study).

A total of 74 participants were identified and approached to participate in the study, of whom 46 consented (Figure 2). In total, 28 participants declined because they were not interested in participating or for personal reasons. A total of 15 participants were initially allocated to each of the three conditions; 3 participants withdrew from the study. One participant in the VR group was admitted to the hospital after completion of the baseline measures and was unable to continue upon their return. A second participant in the active control withdrew after the baseline measures, as they did not wish to continue. A final participant in the passive control withdrew when follow-up measures began, as they did not wish to continue. As this was a per-protocol analysis, the participant in the VR group was replaced with another participant; however, COVID-19 restrictions prevented the replacement of participants in the other two conditions because of site access restrictions during the research period.

The background characteristics and baseline results of the outcome measures of participants who completed the study are reported in Table 1. Using a cutoff score of 37.5 for the AES [3,71], 65% (28/43) of participants met the criteria for a diagnosis of apathy at baseline. Of the remaining participants, 7% (3/43) scored 37, 12% (5/43) scored between 33 and 36, and 16% (7/43) scored between 22 and 28.

**Table 1:** Participant demographic and clinical characteristics by study group at baseline (N=43).

Participant characteristics		Virtual reality (n=15)	Active control (n=14)	Passive control (n=14)	Total (n=43)
Age (years), mean (SD; range)		81.7 (6.6; 72-93)	85.9 (8.1; 71-95)	87 (8.7; 73-103)	84.8 (8.0; 71-103)
Sex (female), n (%)		10 (67)	9 (64)	9 (64)	28 (65)
Years of education, mean (SD)		11.7 (3.1)	10.7 (2.6)	11.0 (4.6)	11.2 (3.4)
**Marital status, n (%)**					
	Married	3 (20)	2 (14)	5 (36)	10 (23)
	Divorced or widowed	11 (73)	11 (79)	8 (57)	30 (70)
	Single	1 (7)	1 (7)	1 (7)	3 (7)
Depression and anxiety medication, n (%)		5 (33)	3 (21)	8 (57)	16 (37)
**Primary diagnosis, n (%)**					
	Memory-related, dementia, or Parkinson disease	5 (33)	3 (21)	3 (21)	11 (26)
	Heart disease	8 (53)	4 (29)	8 (57)	20 (47)
	Stroke	2 (13)	2 (14)	2 (14)	6 (14)
	Other	0 (0)	5 (36)	1 (7)	6 (14)
**Outcome measures at baseline, mean (SD)**					
	Apathy Evaluation Scale	35.3 (8.7)	41.8 (7.1)	44.3 (9.5)	40.3 (9.1)
	Geriatric Depression Scale	4.1 (3.6)	3.5 (2.5)	4.4 (3.0)	4.0 (3.0)
	ACE-III^a^	72.9 (17.7)	71.2 (13.7)	72.2 (13.6)	72.1 (14.9)
	QOL-AD^b^	35.4 (5.8)	36.1 (5.2)	32.9 (6.0)	34.8 (5.7)
	Three-Item Loneliness Scale	4.5 (1.5)	4.2 (1.4)	5.0 (2.5)	4.6 (1.9)

^a^ACE-III: Addenbrooke Cognitive Examination III.

^b^QOL-AD: Quality of Life in Alzheimer Disease.

### Primary Outcome

For contrast 1 (pooled VR and active control groups compared with passive control), there was a significant main effect of group, with higher apathy scores in the passive control group. For contrast 2 (VR group compared with active control), the main effect of group was not significant (Table 2).

**Table 2:** Fixed effects parameter estimates for the Apathy Evaluation Scale.

Parameter			Estimate (SE; 95% CI)		*t* statistic (*df*)		*P* value		Partial *η^2^*
**Main effect**									
	Time	−0.54 (0.78; −2.06 to 0.98)		−0.69 (40)		.49		0.012	
	Contrast 1^a^: passive versus (active and VR^b^)	5.60 (2.55; 0.61 to 10.59)		2.20 (40)		*.03^c^*		0.108	
	Contrast 2^d^: active versus VR	5.33 (2.91; −0.36 to 11.03)		1.84 (40)		.07		0.078	
**Interaction**									
	Contrast 1: time×passive versus (active and VR)	−0.26 (1.66; −3.51 to 2.99)		−0.16 (40)		.88		0.001	
	Contrast 2: time×active versus VR	−2.24 (1.89; −5.95 to 1.47)		−1.18 (40)		.24		0.034	

^a^Contrast 1 compares the pooled interventions (virtual reality and active control) with the passive control group.

^b^VR: virtual reality.

^c^Italics indicates significant values.

^d^Contrast 2 compares both intervention groups (virtual reality and active control).

Contrast 1 (pooled VR and active control groups compared with passive control) revealed that an intervention did not significantly change AES scores from baseline to follow-up (ie, the interaction between group and time was not significant). Contrast 2 (VR group compared with active control) was also not significant (Table 2). The addition of covariates (cognition and depression) did not change the statistical significance.

When restricting the sample to the subgroup that met the AES cutoff of 37.5, the same pattern of results was found (Table 3). The sample size was reduced to 28 (7 in the VR group, 10 in the active control group, and 11 in the passive control group).

**Figure 3:**
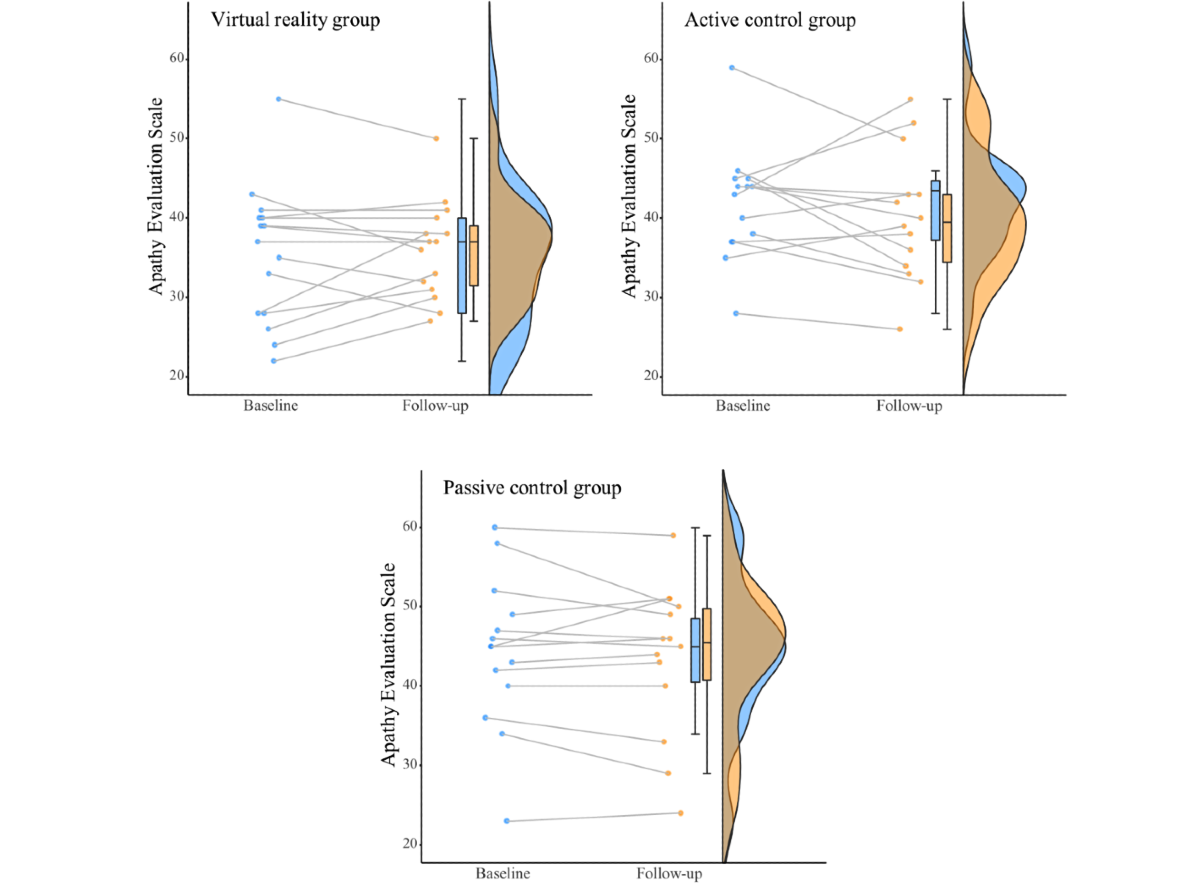
Baseline and follow-up apathy scores for each group with each line representing one participant. Boxplots and rainclouds indicate distribution in baseline and follow-up scores.

Figure 3 presents the baseline and follow-up AES scores for the participants in each group.

**Table 3:** Fixed effects parameter estimates for the Apathy Evaluation Scale for subgroup meeting the Apathy Evaluation Scale cutoff of 37.5 at baseline.

Parameter		Estimate (SE; 95% CI)	*t* statistic (*df*)	*P* value	Partial *η^2^*
**Main effect**					
	Time	−1.38 (1.01; −3.36 to 0.60)	−1.36 (25)	.19	0.069
	Contrast 1^a^: passive versus (active and VR^b^)	5.12 (2.10; 1.01 to 9.23)	2.44 (25)	*.02^c^*	0.193
	Contrast 2^d^: active versus VR	2.30 (2.65; −2.90 to 7.50)	0.87 (25)	.39	0.029
**Interaction**					
	Contrast 1: time×passive versus (active and VR)	1.66 (2.04; −2.35 to 5.66)	0.81 (25)	.43	0.026
	Contrast 2: time×active versus VR	−0.14 (2.58; −5.21 to 4.92)	−0.06 (25)	.96	<0.001

^a^Contrast 1 compares the pooled interventions (virtual reality and active control) with the passive control group.

^b^VR: virtual reality.

^c^Italics indicates significant values.

^d^Contrast 2 compares both intervention groups (virtual reality and active control).

### Secondary Outcomes

No significant results were observed for the ACE-III and GDS (Table S1 in Multimedia Appendix 1). Table S2 in Multimedia Appendix 1 presents results restricting the sample to the subgroup that met the AES cutoff of 37.5 at baseline.

### Exploratory Outcomes

No significant results were observed for the QOL-AD scale and the Three-Item Loneliness Scale (Table S1 in Multimedia Appendix 1). Table S2 in Multimedia Appendix 1 presents results restricting the sample to the subgroup that met the AES cutoff of 37.5 at baseline.

#### SSQ Results

The average pre-VR SSQ scores and post-VR SSQ scores for sessions 1 and 3 were compared. The results were not statistically significant (Table 4). There were no dropouts because of side effects from the HMD use. Two participants reported back the after effects that occurred in the evening after their first morning VR session. This included a headache in one participant and a heavy-head feeling in another participant. The after effects were short-lived and did not cause significant discomfort.

**Table 4:** SSQ^a^ means and SDs with statistics and effect size.

SSQ	Pre-SSQ, mean (SD)	Post-SSQ, mean (SD)	Statistic	*P* value	*d* value
Total	4.86 (6.11)	5.49 (5.98)	6.50	.46	−0.38
Nausea	5.72 (7.90)	6.36 (10.30)	5.50	.68	−0.27
Oculomotor	5.56 (7.14)	7.83 (9.11)	4.50	.25	−0.57
Disorientation	5.10 (10.01)	5.57 (8.40)	3.50	.71	−0.30

^a^SSQ: Simulator Sickness Questionnaire.

#### Staff Questionnaire

The Staff Questionnaire data were not included in the analysis. This was because of missing data for 12 participants caused by COVID-19 restrictions that did not allow follow-up with staff. Therefore, this would have made the results unreliable.

#### Session Record

The sum of the three sessions was used; therefore, the scores ranged from 0 to 9 for each subscale. The total score of the combined subscales ranged from 0 to 45 for all three sessions. Results comparing the VR group with active control for total and subscale scores were not statistically significant (Table 5). Results from the questions asked in the session record found that 73% (11/15) of participants in the VR group preferred viewing content in VR to a flat screen if given a choice, 7% (1/15) stated that they preferred a flat screen to VR, and 20% (3/15) were undecided. The proportion of participants who responded a preference for VR was significant (*Χ*^2^_2_=11.2; *P*=.004). Participants in both the VR and active control groups stated that they would like to do reminiscence again.

**Table 5:** Session record means and SDs for each group with statistics and effect size.

Session record	Virtual reality group, mean (SD)	Active control, mean (SD)	Statistic	*P* value	*d* value
Total	36.40 (7.21)	38.86 (5.02)	84.0	.37	0.20
Attendance	7.93 (1.44)	8.14 (1.61)	94.5	.60	0.10
Memory	5.60 (2.90)	7.36 (2.06)	66.0	.09	0.37
Interaction	8.20 (1.26)	8.50 (0.76)	99.5	.80	0.05
Responsiveness	7.53 (1.85)	8.57 (1.09)	72.0	.08	0.31
Enjoyment	7.20 (1.42)	6.29 (1.33)	68.5	.10	0.35

## Discussion

### Summary

To the best of our knowledge, this is the first clinical trial to compare the use of VR with flat screen technology for reminiscence in an aged care setting with a usual care control group. Well-established and validated measures were used, and there was a 100% adherence rate once the intervention commenced. It was also found that with correct procedures, side effects of using HMDs can be minimized and that VR technology can be implemented in an aged care setting. Participants also enjoyed the process of reminiscing indicated by the session record with all participants stating that they would like to do reminiscence again.

### Content

It was found that the content for both intervention groups for all three sessions could be sourced using readily available apps, as per the feasibility study [72]. Common types of content the participants viewed included their original family home, the school they attended, places of employment, and travel destinations. In many instances, it was possible to tour inside buildings or places of interest and remote tourist destinations because of the increased access available in both the Wander app for the VR group and Google Street View for the active control group. Both YouTube and YouTube VR also provided access to travel destinations in addition to the music that the participant had memories of.

### VR Group and Active Control Comparison

The results of this study are inconclusive, with no significant differences found between the intervention groups. Median scores for apathy decreased in the active control group and remained the same in the VR group from baseline to follow-up (Figure 3). Results from the session record for the VR group indicated that there was a preference for viewing content in VR than on a flat screen. In addition, a nonsignificant small effect size was found for enjoyment with higher scores in the VR group; however, similar small effect sizes with higher scores in the active control group were reported for responsiveness and memory. Anecdotally, there were more positive emotions observed in participants in the VR group when participants viewed familiar content compared with the active control group. The enjoyment or preference for VR did not translate to improved outcomes over flat screen technology. The findings from our study are consistent with those of a previous study [73] examining the use of VR by older adults with dementia, where no significant changes were found in outcome measures; however, VR did provide an engaging experience from the perspective of both participants and caregivers.

The availability of content when using the apps in the VR group (Wander and YouTube VR) was equivalent to that of the apps used for the active control group (Google Street View and YouTube); however, there were instances where the internet in general was used for the active control group, increasing the availability of content. In addition, in the active control, physical items were used in some instances (photo albums) if the participant requested this; however, the laptop was still always used, and the physical items only supplemented the reminiscence experience. This means that there were differences other than the display technology between the two intervention groups.

In both YouTube VR and Wander, participants had 360° vision by turning in the swivel chair and if the researcher used the controller to turn in the virtual environment. The Wander app provided increased movement backward and forward through the environment navigated by the researcher while participants remained stationary. The Oculus Quest HMD used in this study is capable of six degrees of freedom (head and body movement). As the participants were stationary and the apps used did not use the full capabilities of movement in the virtual environment, the participants were not provided with the optimum VR experience. Interactive VR with software using six degrees of freedom where the participant moves in the virtual environment has been found to increase the sense of presence and positive affect [34], which may not be an option for older adults in residential aged care because of safety aspects, including increased risk of falling. However, research using HMDs limited to three degrees of freedom (head movement only) for pain and posttraumatic stress disorder has reported positive outcomes [36,74].

Although VR provides increased immersion and realism, the interaction between the participant and researcher or therapist can be compromised by the HMD. Improving outcomes related to neuropsychiatric symptoms or quality of life may be influenced by interactions with the researcher or therapist. For example, reminiscence therapy traditionally involves attentive behaviors, including eye contact and body language [75]. Wearing the HMD during the interventions did not provide the same level of personal interaction as in the active control group. This may explain why VR in this study did not demonstrate the same positive outcomes seen in VR research using exposure and distraction-based therapies [35,36], where there is increased focus on the content being viewed rather than interaction with the therapist.

It is important not to discount the possibility that immediate effects provide enjoyment and stimulation during an intervention for participants that may not translate to longer-term measures [76]. Positive results related to apathy and emotion reported in previous studies using HMDs have been measured directly after a VR experience [46,72,77]. What has not been reported in this study is any immediate physiological effect when taking measures directly after the VR experience and how this compares with flat screen technology.

### Effect of Reminiscence Therapy

There were no significant differences in outcomes from baseline to follow-up between the pooled intervention and passive control groups. These results contrast with the participant enthusiasm and enjoyment during the intervention in both groups evidenced by 100% participation once commencing, measured enjoyment levels from the session record, wanting to participate in reminiscence again, and anecdotal feedback from staff. Consistent with the meta-analysis by Pinquart and Forstmeier [18], a medium effect size was observed for depression, with reduced depression in the intervention groups over time, but this effect was not statistically significant (*P*=.09; partial η^2^=0.069; Table S1 in Multimedia Appendix 1).

Compared with previous traditional reminiscence studies including apathy as an outcome, our study did have a relatively small dosage and was conducted over a short period [78,79], which may have contributed to the lack of statistically significant results. However, in an aged care setting, it may not be necessary to see improvement but a reduction or stabilization in the rate of decline of conditions, including apathy for the intervention to benefit participants [80]. Entry into aged care facilities results in accelerated cognitive decline compared with people living in the community [81]. It is unknown whether the intervention in this study was successful in altering the trajectory of decline from apathy to more severe symptoms that would require a longer intervention period. The number of sessions was selected to increase the feasibility of the trial, reduce attrition bias in an aged care context, and determine from immediate effects reported from our feasibility study [72]. Dose-effect models do have a nonlinear relationship [82], and previous studies have seen improvements in three sessions using reminiscence to improve agitation [83], verbal fluency and communication [84], increased engagement [85], self-esteem and depression [86,87], and anxiety [88]. As VR can provide increased stimulation compared with traditional therapies, the dosage required is yet to be established. There was also a significant difference between the groups at baseline, which may have also contributed to the lack of statistical differences.

The pooled scores of the intervention groups on apathy were significantly lower at baseline compared with those of the passive control group and may have accounted for a lack of treatment effect. Equality between groups is more likely to be difficult to achieve in smaller samples [89], particularly if the sample includes older adults [90]. In an aged care setting, this is further complicated because of the focus on providing support for people to age in their homes. This means that residents entering aged care are now older, have complex needs, and are taking multiple medications [91]. Although participants with severe cognitive decline were excluded and covariates controlled for baseline differences, there were day-to-day differences in a participant’s condition commonly observed throughout the research period. Despite the use of reliable and valid measures, the selection of outcome measures is a challenging issue in residential aged care [92], and they may not be sensitive to changes in all residents at all times.

### VR Side Effects

Results from SSQ revealed negligible to minimal side effects according to the cutoff scores [93]. There were 40% (6/15) of participants who reported symptoms before VR because of existing conditions. Therefore, it is important to take measures before exposure of this population to VR to accurately understand and differentiate symptoms occurring during HMD use. Two participants reported possible after effects in the VR group. This occurred in the evening after a morning VR session in 2 participants, one participant reported a headache and the second had a heavy-head feeling. The symptoms were not long-lasting and did not cause any significant discomfort. A consideration for future research is to advise the participants and staff that these symptoms may occur.

### Limitations

Participants in this study were not classified as having apathy at baseline according to the AES; therefore, there were participants in the study who had relatively low scores at baseline, leaving little room for improvement. However, this was not supported by subgroup analysis. The approach of not using a cutoff score to classify participants as having apathy was taken because of positive changes seen in our feasibility study [72] in participants with low apathy scores below recommended cutoffs, and the high prevalence of apathy in aged care.

The study included participants with different conditions who were taking a range of medications, and the sample size was relatively small; these factors affected generalizability and was also a possible reason for the lack of significant differences in outcome measures over time. In addition, only those interested in reminiscing may have participated in the research in the intervention groups. Participant selection for recruitment was performed by staff at the aged care facility. This may have resulted in selection bias within the sample. Although 3 separate sites were used in this study, they were run by the same aged care facility, therefore providing a similar quality of care and within a 20-km radius of each other. To maximize comparability between participants, a cutoff score on the Psychogeriatric Assessment Scale was used; therefore, participants with severe cognitive impairment were not included in the trial. Maximizing comparability further would have severely restricted recruitment numbers and the feasibility of the trial. This was overcome by including covariates at baseline in the final analyses, which did not significantly affect the results. As participants with severe cognitive impairment were excluded from this study, we cannot make any assumptions as to whether using HMD is suitable for this stage of cognitive impairment. A sensory approach to reminiscence using physical objects or items, including pictures, may be the best approach for those with severe cognitive impairment [94].

The results of this study may have been affected by the global pandemic. Additional hygiene measures introduced during the research period included wearing masks. Many participants in this age group and setting can have hearing problems, and wearing a mask reduces the communication of facial expressions and reduces the projection of a person’s voice and clarity. How this influenced both outcome measures and intervention sessions was not known. Apathy has been reported as one of the most affected symptoms in patients with Alzheimer disease [95] and increased caregiver burden in patients with neuropsychiatric symptoms [96] during the pandemic. Participants also had severely restricted visitations from family and friends at times during the research period, which may have exacerbated the symptoms and reduced the likelihood of statistically significant outcomes of the intervention.

### Conclusions

This study examined changes in apathy using VR. Although no statistically significant difference was found between the VR group and the active control group, we have demonstrated that VR can be implemented in an aged care setting with correct protocols in place and that residents in aged care enjoy the process of reminiscing. The use of VR provides access to a wide range of content that is always increasing, and aged care facilities may be able to use VR in other contexts, for example, in lifestyle activities for music or travel.

To facilitate implementation in residential aged care, potential co-designers of VR activities could consist of key stakeholders, including management of aged care facilities, staff who organize and deliver activities, residents, and family or friends of residents. Staff members who deliver activities in residential aged care facilities would normally already have the necessary skills for dealing with and engaging with people in residential aged care. Clinician involvement in training in the use of HMDs would assist in managing health and safety risks and providing the best experience for both implementing it as an activity in residential aged care and for optimum data collection in research. This would include recommendations of HMD type, monitoring of side effects, correct fitment and setup of HMDs, and sourcing of content.

The success of using VR in exposure- and distraction-based therapies may not transfer to therapeutic uses in an aged care setting. However, the results from this trial are not definitive, and longer-term research including more sessions is required. Giving participants a choice between immersive and flat screen technology can assist in increasing enjoyment and participation rates in lifestyle activities, ultimately providing more engaged residents for aged care facilities, particularly for those with apathy. Physiological measures for assessing immediate effects and the use of actigraphy as an objective measure of apathy are an avenue for further research that will be reported from this study.

## Appendix

Multimedia Appendix 1 Secondary and exploratory results.

## Abbreviations

ACE-III: Addenbrooke Cognitive Examination III
AES: Apathy Evaluation Scale
CONSORT: Consolidated Standards of Reporting Trials
GDS: Geriatric Depression Scale
HMD: head-mounted displays
QOL-AD: Quality of Life in Alzheimer Disease
SSQ: Simulator Sickness Questionnaire
VR: virtual reality
