# Supplementary material for: The Effect of Reminiscence Therapy Using Virtual Reality on Apathy in Residential Aged Care: Multisite Nonrandomized Controlled Trial
Source: J Med Internet Res. 2021 Sep 20;23(9):e29210. doi: 10.2196/29210 (PMC8491119; doi:10.2196/29210)
Supplement: Multimedia Appendix 1 [file jmir_v23i9e29210_app1.docx]

**Multimedia Appendix 1. Secondary and exploratory results.**

**Table S1.** Fixed effects parameter estimates for secondary and exploratory outcomes.

| Parameter | Estimate | SE | 95% CI | *t* statistic (df) | *P* value | partial η^2^ |
| --- | --- | --- | --- | --- | --- | --- |
| **Main Effect - ACE-III**^a^ |  |  |  |  |  |  |
| Time | 1.09 | 0.90 | -0.67 to 2.85 | 1.22 (40) | .23 | 0.036 |
| Contrast 1^b^: passive vs (active and VR^c^) | 1.44 | 5.01 | -8.37 to 11.26 | 0.29 (40) | .78 | 0.002 |
| Contrast 2^d^: active vs VR | -2.54 | 5.72 | -13.75 to 8.67 | -0.44 (40) | .66 | 0.005 |
| **Interaction - ACE-III** |  |  |  |  |  |  |
| Contrast 1: time × passive vs (active and VR) | 2.54 | 1.91 | -1.21 to 6.29 | 1.33 (40) | .19 | 0.042 |
| Contrast 2: time × active vs VR | -1.78 | 2.18 | -6.06 to 2.50 | -0.81 (40) | .42 | 0.016 |
| **Main Effect – GDS**^e^ |  |  |  |  |  |  |
| Time | -0.02 | 0.30 | -0.60 to 0.57 | -0.06 (40) | .95 | <0.001 |
| Contrast 1: passive vs (active and VR) | 1.09 | 1.01 | -0.89 to 3.06 | 1.08 (40) | .29 | 0.028 |
| Contrast 2: active vs VR | -0.75 | 1.15 | -3.00 to 1.50 | -0.65 (40) | .52 | 0.011 |
| **Interaction - GDS** |  |  |  |  |  |  |
| Contrast 1: time × passive vs (active and VR) | 1.10 | 0.64 | -0.15 to 2.35 | 1.72 (40) | .09 | 0.069 |
| Contrast 2: time × active vs VR | -0.23 | 0.73 | -1.66 to 1.19 | -0.32 (40) | .75 | 0.003 |
| **Main Effect – QOL-AD**^f^ |  |  |  |  |  |  |
| Time | -0.09 | 0.58 | -1.23 to 1.06 | -0.15 (40) | .88 | 0.001 |
| Contrast 1: passive vs (active and VR) | -2.64 | 1.75 | -6.07 to 0.80 | -1.50 (40) | .14 | 0.054 |
| Contrast 2: active vs VR | 1.87 | 2.00 | -2.05 to 5.79 | 0.94 (40) | .36 | 0.021 |
| **Interaction – QOL-AD** |  |  |  |  |  |  |
| Contrast 1: time × passive vs (active and VR) | 0.56 | 1.24 | -1.88 to 2.99 | 0.45 (40) | .66 | 0.005 |
| Contrast 2: time × active vs VR | 2.26 | 1.42 | -0.52 to 5.04 | 1.59 (40) | .12 | 0.060 |
| **Main Effect – Loneliness**^g^ |  |  |  |  |  |  |
| Time | -0.15 | 0.20 | -0.54 to 0.24 | -0.74 (40) | .46 | 0.014 |
| Contrast 1: passive vs (active and VR) | 0.66 | 0.53 | -0.38 to 1.70 | 1.25 (40) | .22 | 0.038 |
| Contrast 2: active vs VR | -0.60 | 0.61 | -1.79 to 0.58 | -1.00 (40) | .33 | 0.024 |
| **Interaction - Loneliness** |  |  |  |  |  |  |
| Contrast 1: time × passive vs (active and VR) | 0.01 | 0.42 | -0.82 to 0.84 | 0.02 (40) | .99 | <0.001 |
| Contrast 2: time × active vs VR | -0.70 | 0.48 | -1.65 to 0.25 | -1.45 (40) | .16 | 0.050 |

^a^ACE-III: Addenbrooke Cognitive Examination III.

^b^Contrast 1 compares the pooled interventions (virtual reality and active control) with the passive control group.

^c^VR: virtual reality.

^d^Contrast 2 compares both intervention groups (virtual reality and active control).

^e^GDS: Geriatric Depression Scale.

^f^QOL-AD: Quality of Life in Alzheimer Disease.

^g^Loneliness: Three-Item Loneliness Scale.

**Table S2.** Fixed effects parameter estimates for secondary and exploratory outcomes including subgroup meeting Apathy Evaluation Scale cutoff of 37.5 at baseline.

| Parameter | Estimate | SE | 95% CI | *t* statistic (df) | *P* value | partial η^2^ |
| --- | --- | --- | --- | --- | --- | --- |
| **Main Effect – ACE-III**^a^ |  |  |  |  |  |  |
| Time | 0.44 | 1.20 | -1.91 to 2.80 | 0.37 (25) | .72 | 0.005 |
| Contrast 1^b^: passive vs (active and VR^c^) | 3.05 | 5.77 | -8.25 to 14.35 | 0.53 (25) | .60 | 0.011 |
| Contrast 2^d^: active vs VR | 4.77 | 7.30 | -9.53 to 19.08 | 0.65 (25) | .52 | 0.017 |
| **Interaction – ACE-III** |  |  |  |  |  |  |
| Contrast 1: time × passive vs (active and VR) | 2.74 | 2.43 | -2.02 to 7.50 | 1.13 (25) | .27 | 0.049 |
| Contrast 2: time × active vs VR | -2.66 | 3.07 | -8.68 to 3.37 | -0.86 (25) | .40 | 0.029 |
| **Main Effect – GDS**^e^ |  |  |  |  |  |  |
| Time | 0.14 | 0.42 | -0.69 to 0.96 | 0.32 (25) | .75 | 0.004 |
| Contrast 1: passive vs (active and VR) | -0.17 | 1.16 | -2.44 to 2.11 | -0.14 (25) | .89 | 0.001 |
| Contrast 2: active vs VR | -3.59 | 1.47 | -6.47 to -0.71 | -2.45 (25) | *.02*^f^ | 0.193 |
| **Interaction - GDS** |  |  |  |  |  |  |
| Contrast 1: time × passive vs (active and VR) | 1.16 | 0.85 | -0.51 to 2.83 | 1.36 (25) | .19 | 0.069 |
| Contrast 2: time × active vs VR | -0.50 | 1.08 | -2.61 to 1.61 | -0.46 (25) | .65 | 0.009 |
| **Main Effect – QOL-AD**^g^ |  |  |  |  |  |  |
| Time | 0.09 | 0.74 | -1.35 to 1.53 | 0.13 (25) | .90 | 0.001 |
| Contrast 1: passive vs (active and VR) | -1.04 | 2.01 | -4.98 to 2.90 | -0.52 (25) | .61 | 0.011 |
| Contrast 2: active vs VR | 5.22 | 2.54 | 0.24 to 10.21 | 2.05 (25) | .05 | 0.144 |
| **Interaction – QOL-AD** |  |  |  |  |  |  |
| Contrast 1: time × passive vs (active and VR) | -0.69 | 1.49 | -3.60 to 2.23 | -0.46 (25) | .65 | 0.008 |
| Contrast 2: time × active vs VR | 2.36 | 1.88 | -1.33 to 6.04 | 1.25 (25) | .22 | 0.059 |
| **Main Effect – Loneliness**^h^ |  |  |  |  |  |  |
| Time | 0.04 | 0.27 | -0.49 to 0.56 | 0.13 (25) | .90 | 0.001 |
| Contrast 1: passive vs (active and VR) | -0.21 | 0.65 | -1.49 to 1.08 | -0.31 (25) | .76 | 0.004 |
| Contrast 2: active vs VR | -1.69 | 0.83 | -3.32 to -0.07 | -2.05 (25) | .05 | 0.143 |
| **Interaction - Loneliness** |  |  |  |  |  |  |
| Contrast 1: time × passive vs (active and VR) | 0.08 | 0.54 | -0.98 to 1.15 | 0.16 (25) | .88 | 0.001 |
| Contrast 2: time × active vs VR | -1.41 | 0.69 | -2.76 to -0.07 | -2.06 (25) | .05 | 0.145 |

^a^ACE-III: Addenbrooke Cognitive Examination III.

^b^Contrast 1 compares the pooled interventions (virtual reality and active control) with the passive control group.

^c^VR: virtual reality.

^d^Contrast 2 compares both intervention groups (virtual reality and active control).

^e^GDS: Geriatric Depression Scale.

^f^Significant values in italics.

^g^QOL-AD: Quality of Life in Alzheimer Disease.

^h^Loneliness: Three-Item Loneliness Scale.
